# Partially Defatted *Hermetia illucens* Larva Meal in Diet of Eurasian Perch (*Perca fluviatilis*) Juveniles

**DOI:** 10.3390/ani10101876

**Published:** 2020-10-14

**Authors:** Vlastimil Stejskal, Hung Quang Tran, Marketa Prokesova, Tatyana Gebauer, Pham Thai Giang, Francesco Gai, Laura Gasco

**Affiliations:** 1 South Bohemian Research Center of Aquaculture and Biodiversity of Hydrocenoses, Faculty of Fisheries and Protection of Waters, Institute of Aquaculture and Water Protection, University of South Bohemia in Ceske Budejovice, Husova tř. 458/102, 370 05 České Budějovice, Czech Republic; stejskal@frov.jcu.cz (V.S.); htranquang@frov.jcu.cz (H.Q.T.); mprokesova@frov.jcu.cz (M.P.); tvanina@frov.jcu.cz (T.G.); 2 Research Institute for Aquaculture No. 1, Dinh Bang, Bac Ninh, Tu Son, Vietnam; ptgiang@ria1.org; 3 National Research Council, Institute of Sciences of Food Production, Largo P. Braccini 2, 10095 Grugliasco, Italy; 4 Department of Agricultural, Forest and Food Sciences, University of Torino, Largo P. Braccini 2, 10095 Grugliasco, Italy; laura.gasco@unito.it

**Keywords:** alternative feed, insect meal, splenic lipidosis, economic and environmental sustainability

## Abstract

**Simple Summary:**

The replacement of fishmeal by insect meal is a promising strategy to obtain more sustainable fish feeds, a major goal in aquaculture. Black soldier fly *Hermetia illucens* larva meal has a high crude protein and fat content, essential for omnivorous and carnivorous fish. We used partially defatted *H. illucens* larva meal as a substitute for 20%, 40% and 60% of the fishmeal in standard diets for Eurasian perch and measured its effect on growth performance, feed utilization, body indices, fish body composition and blood indices. We found no significant differences in survival, size heterogeneity, hematology indices; or in whole-body dry matter, crude protein and ether extract content. The 60% inclusion reduced final body weight, specific growth rate, feeding rate, protein efficiency ratio, condition factor and hepatosomatic index. The fish-in-fish-out index decreased proportionally with increased *H. illucens* meal inclusion. Partially defatted *H. illucens* larva meal seems to be a promising alternative to fishmeal for Eurasian perch nutrition at moderate inclusion level.

**Abstract:**

Insect meal is gaining increased attention in aquafeed formulations due to high protein content and an essential amino acid profile similar to that of fishmeal. To investigate insect meal in feed for European perch *Perca fluviatilis*, a promising candidate for European intensive culture, we replaced standard fishmeal with partially defatted black soldier fly *Hermetia illucens* larva meal at rates of 0%, 20%, 40% and 60% (groups CON, H20, H40 and H60, respectively) and compared growth performance, somatic indices, hematological parameters, whole-body proximate composition and occurrence of spleen lipidosis. In addition, we assessed the economic and environmental sustainability of the tested feeds by calculating economic conversion ratio (ECR) and economic profit index (EPI). The tested groups did not differ in survival rate. Significant differences were documented in final body weight and specific growth rate, with the highest values in CON, H20 and H40. The proximate composition of fish whole-body at the end of the experiment did not differ in dry matter, crude protein or ether extract, while organic matter, ash and gross energy composition showed significant differences. The fatty acid content and n-3/n-6 ratio showed a decreasing trend with increasing H. *illucens* larva meal inclusion. No differences were found in hematological parameters among tested groups. The H. *illucens* larva meal inclusion significantly affected ECR and EPI, even at 20% inclusion level the cost of diets did not differ from the control fish meal based diet. Results suggested that 40% inclusion of H. *illucens* larva meal can be used successfully in standard diets for perch.

## 1. Introduction

Intensive culture of the carnivorous freshwater Eurasian perch (*Perca fluviatilis* L.) is increasing in recirculating aquaculture systems (RAS) and represents an expanding branch of commercial fish farming in Europe. Nevertheless, as a relatively new aquaculture species, production is low [1]. It is commonly reared on feed formulated primarily for salmonids or marine fish species [2]. Diets for carnivorous species contain high levels of protein, which have been obtained from marine fishmeal (FM), considered optimal because of its balanced nutritional composition [3,4]. Currently, with FM increased cost and unsustainability [5], plant protein sources, especially soybean meal, are being used in aquaculture to decrease the dependency on FM and reduce feed costs [4]. High levels of plant protein in feeds can reduce growth performance or induce fish health issues, chiefly due to imbalance in essential amino acid (EAA) content, low feed acceptance and the presence of anti-nutritional factors [3,6,7]. Processed animal proteins (PAP) such as poultry by-product meal, meat meal and meat and bone meal are valid proteins for aquaculture feeds but their use is limited by legislation. In the EU, PAP from poultry and swine have only recently been reintroduced into aquafeed (EC No. 56/2013) after more than 10 years of ban due to Bovine Spongiform Encephalopathy (EC No 999/2001), while in other parts of the world, its use is common practice [8].

Recently, interest has turned to PAPs from insects as a component of aquafeeds [9,10]. Insect larva meals are rich in proteins and their EAA profile is close to that of FM and considered superior to that of plant proteins [9]. The use of insect PAP has recently been sanctioned by the European Commission (Brussels, Belgium) (Regulation 2017/893/EC, 2017).

The black soldier fly *Hermetia illucens* belongs to the family *Stratiomyidae* and is among the most promising insect species for mass-rearing for animal feed [11]. Commercial *H. illucens* meal has an average protein content of 55% dry matter (DM) with lipid content ranging from 5% to 35% DM, depending on the defatting process applied during meal production. Research into its efficacy has thus far been contradictory: Similar or better growth performance to that of fish fed conventional protein sources (mainly FM or soybean meal) using commercial *H. illucens* meal at inclusion levels from 2.5% to 40% were obtained for Atlantic salmon (*Salmo salar)* [12,13,14], rainbow trout (*Oncorhynchus mykiss)* [15,16], European sea bass (*Dicentrarchus labrax)* [17], yellow catfish (*Pelteobagrus fulvidraco)* [18] and rice field eel (*Monopterus albus)* [19]. Conversely, other authors reported reduced acceptance and growth [20,21], with high levels of inclusion. Divergence in results is likely due to the differences among H. *illucens* meals and the level of inclusion in the diet and also suggest species differences in adaptation to insect meals. The use of *H. illucens* meal in perch diets has not been investigated.

The goal of this research was to determine the effects of partially defatted *H. illucens* meal as partial substitute for FM on growth performance, somatic indices, occurrence of splenic lipidosis, hematological parameters and proximate whole-body composition of juvenile *P. fluviatilis*. The research also aimed to provide new data on the economic and environmental sustainability of this novel protein source.

## 2. Materials and Methods

An 84-day growth trial was carried out at the Faculty of Fisheries and Water Protection of the University of South Bohemia (České Budějovice, Czech Republic). The trial was designed and carried out in accordance with the Czech and European Communities Directive (2010/63/EU) on the protection of animals used for scientific purposes, protocol number MSMT-6744/2018-2.

### 2.1. Experimental Diets

Four experimental diets were formulated to be isonitrogenous (crude protein, CP: ~54 g 100 g DM); isolipidic (ether extract, EE: ~13 g 100 g DM); and isoenergetic (gross energy, GE: ~23 MJ kg DM). An FM-based diet was used as control (CON) and three additional diets included FM replacement with 20% (H20), 40% (H40) and 60% (H60) partially defatted H. *illucens* larva meal obtained with a mechanical process performed using high pressure and without solvents was provided by Hermetia Deutschland GmbH & Co. KG (Baruth/Mark, Germany). In order to ensure that diets were isonitrogenous, isolipidic and isoenergetic, the proportion of wheat meal and fish oil was reduced with increase in *H. illucens*.

The experimental feeds were prepared at the Department of Agricultural, Forest and Food Sciences experimental facility. Finely ground ingredients and fish oil were thoroughly mixed with water and pelleted using a 2 mm meat grinder and dried at 50 °C for 48 h. Feeds were stored in dark bags at −20 °C until use. The ingredients of the experimental diets are reported in Table 1. An additional control group (BIO) was fed a commercial extruded diet (BioMar Inicio 2 mm, BioMar A/S, Brande, Denmark) containing fish meal, wheat gluten, wheat, pea protein, soybean concentrate, rapeseed oil, fish oil and yeast extract as main ingredients. Proximate composition (on a wet basis) according to manufacturer’s label was CP 52%, crude lipid 23%, carbohydrates 12%, ash 8.7%, fiber 0.9%, total phosphorus (P) 1.2% and GE 23.5 MJ/kg.

### 2.2. Fish and Feeding Trial

Eurasian perch juveniles were obtained from pond-reared larvae and intensively reared juveniles in an RAS [22]. The RAS (4360 L total water volume) included fifteen 75 L rearing tanks, a mechanical drum filter AEM 15 (AEM-Products V.O.F., Lienden, The Netherlands), a 1620 L tank with a series of filtration sections, Bioakvacit PP10 (Jezírka Banát s.r.o., Hněvotín, Czech Republic), a moving bed biofilter (1620 L) with media BT10 (Ratz Aqua & Polymer Technik, Remscheid, Germany), UV treatment AquaForte 55 W (AquaForte, Veghel, The Netherlands) and an Eheim Jäger Thermocontrol 300 flow-through heater (Eheim GmbH & Co KG, Stuttgart, Germany) incorporated directly into the recirculation flow. The flow rate in the tanks was approximately 80 L h^−1^ with light aeration. Photoperiod was set at 12:12 h (dark: light) with light intensity of 500–700 Lx at the surface. Oxygen saturation (83.7 ± 6.2%), pH (6.83 ± 0.52) and water temperature (22.5 ± 0.7 °C) (HACH HQ 40, Germany) were measured daily at 08.00 and 16.00. Ammonia, nitrate and nitrite concentrations were analyzed at two-day intervals with kits (HACH, LCK 304, LCK 339, LCK 341), using a HACH DR2800 spectrophotometer. The concentration of nitrite-N, nitrate-N and ammonia-N were 0.62 ± 0.44 mg L^−1^, 88.88 ± 57.31 mg L^−1^ and 2.07 ± 1.02 mg L^−1^, respectively.

A total of 750 juvenile European perch were lightly anaesthetized (0.3 mL L^−1^ of clove oil), individually weighed (initial body weight (BWi) 21.9 ± 4.2 g) using a digital balance (Pioneer, Ohaus Corporation, Parsippany, NJ, USA, d = 0.01 g) and randomly allocated to one of the fifteen 75 L rectangular plastic tanks at a stocking density of 14.6 kg m^−3^. The four experimental diet groups and the BIO group were randomly allocated to the fifteen tanks, with each diet tested in triplicate. Fish were fed manually to subjectively-judged satiation five times daily (09:00, 11:00, 13:00, 15:00 and 17:00 h). Care was taken to avoid feed waste and to ensure that all supplied feed was consumed. The feeding trial lasted 84 days.

### 2.3. Growth Performance

At the end of the trial, all fish were individually weighed and growth performance was calculated using following equations:

Survival (S, %) = 100 × Nf (Ni − Ns)^−1^

Initial coefficient of variation (ICV, %) = (SD/BWi) × 100

Final coefficient of variation (FCV, %) = (SD/BWf) × 100

Specific growth rate (SGR, % day^−1^) = ((lnBWf − lnBWi)/Nd) × 100

Feed conversion ratio (FCR) = (TFS/WG)

Protein efficiency ratio (PER) = (WG (g)/TPS total protein fed (g, DM))

Feeding rate (FR, %/d) = ((TFS × 100/Nd))/(e (lnBWf + lnBWi) × 0.5),

where Ni and Nf = initial and final number of fish per tank, Ns = number of sampled fish per tank, BWf = final body weight (g), BWi = initial body weight (g), Nd = number of feeding days, TFS = total feed supplied (g), TPS = total protein supplied (g, DM), SD = standard deviation of subsample BW, BWi = initial mean body weight, BWf = final mean body weight, lnBWf = natural logarithm of final body weight, lnBWi = natural logarithm of initial body weight, DM = dry matter, WG = weight gain.

### 2.4. Condition Factor, Somatic Indexes and Occurrence of Spleen Lipidosis

To calculate condition factor (K), at the end of the growth trial, fifty fish from each tank were anaesthetized (0.3 mL L^−1^ of clove oil) and individually weighed and measured for total length (TL, mm) and standard length (SL, mm) within 1 mm using a ruler.

K was calculated as:

K = (BWf/TL) × 100,

where BWf = final body weight (g), TLf = final body length (cm).

At the end of the trial, 30 fish/tank were killed by overdosing of anesthesia with clove oil and wet weight of liver, spleen, viscera and perivisceral fat recorded (±0.01 g) for calculation of somatic indices using the equations:

Hepatosomatic index (HSI) = Wl (weight, g) × 100/BW (body weight, g)

Splenosomatic index (SSI) = Ws (weight, g) × 100/BW (body weight, g)

Viscerosomatic index (VSI) = Wv (weight, g) × 100/BW (body weight, g)

Perivisceral fat index (PFI) = Wpf (weight, g) × 100/BW (body weight, g),

where Wl = liver weight (g), Ws = spleen weight (g), Wv = viscera weight (g), Wpf perivisceral fat weight (g).

Frequency of occurrence splenic lipidosis [2], was calculated according to the equation:

SL = 100/Nt × Nsl,

where Nt is total number of investigated fish and Nsl is number of fish with spleen lipidosis.

### 2.5. Chemical Analyses

The H. *illucens* larva meal chemical analysis was obtained from Renna et al. [15]. The proximate composition and energy of diets are reported in Table 1. Feed samples were finely ground (MLI 204; Bühler AG, Uzwil, Switzerland) and analyzed for DM (AOAC, n. 934.01), CP (AOAC, n. 984.13) and ash (AOAC, n. 942.05) content according to AOAC International [23]. The EE content (AOAC, n. 2003.05) was analyzed according to AOAC International [24]. The GE content was determined using an adiabatic bomb calorimeter (C7000; IKA, Staufen, Germany). Chitin content was determined following Finke [25], by correcting for the amino acid (AA) content of the acid fiber detergent (ADF) fraction and assuming the remainder of the ADF fraction to be chitin. The AA composition of H. *illucens* larva meal and FM used in the experimental diets is shown in Table 2. Amino acid quantification was conducted according to De Marco et al. [26]. After 22 h hydrolysis in 6N HCl at 112 °C under a nitrogen atmosphere, the AA content in the hydrolysate was assessed by HPLC after post-column derivatization. Performic acid oxidation occurred prior to acid hydrolysis for methionine and cystine. Tryptophan was not determined.

At the end of the trial, whole-body homogenate of six fish from each group was analyzed for DM, CP, EE, organic matter (OM) and ash content according to the procedure used for feed analyses [23,24]. The DM content was measured according to AOAC (n. 934.01; [23]).

Fatty acid profiles were determined both in feed and fish whole-body homogenate (3 fish/tank, 9 fish/group), according to the method of Sampels et al. [27]. Initially, lipids were extracted by the hexan-isopropanol method according to Hara and Radin [28]. Fatty acid methyl esters (FAME) were prepared by the BF_3_ method according to Appelqvist [29] and analyzed using FAME C 11:0 as an internal standard by a gas chromatograph (Trace Ultra FID; Thermo Scientific, Milan, Italy) equipped with a flame ionization detector, using a BPX 70 column (length 50 m, i.d. 0.22 mm, film thickness 0.25 μm) (SGE Inc., Austin, TX, USA). The peaks were identified using Thermo Xcalibur 3.0.63 (Thermo Fisher Scientific Inc., Waltham, MA, USA) software and quantification was achieved by comparing sample retention times and peak areas to retention times and peak area in 7 levels (1000 ug/mL–15 ug/mL) of the standard mixture Supelco 37 component FAME mix (Sigma-Aldrich, St. Louis, MO, USA). Fatty acid profiles for feed are shown in Table 3, analysis were performed in triplicate.

### 2.6. Haematological Analyses

At the end of experiment, three fish per tank (nine fish from each group) were over-anaesthetized with clove oil and blood samples were taken for hematological analysis. Red blood cell count (RBCC), hematocrit (HCT), hemoglobin concentration (Hb), mean corpuscular volume (MCV), mean corpuscular hemoglobin (MCH) and mean corpuscular hemoglobin concentration (MCHC) were measured according to Svobodova et al. [30].

### 2.7. Economic Analysis and Environmental Sustainability of Feeds

To determine the relative efficacy and benefits of tested diets, economic conversion ratio (ECR) and economic profit index (EPI) for each tested group was calculated by the following equations:

ECR (€ kg of fish^−1^) = FCR × DP

EPI (€ fish^−1^) = (WG × SP) − (WG × DP),

where FCR is feed conversion ratio (kg feed per kg fish); DP is cost per kg feed; WG is weight gain. The per kilogram cost in euros, excluding labor and taxes, of all components from commercial retailers was as follows: FM = € 1.48; H. *illucens* larva meal = € 3.5; wheat meal = € 0.61; fish oil = € 1.32; gelatinized starch = € 0.75; mineral mixture = € 0.49; vitamin mixture = € 3.85. This resulted in per kg feed cost of CON = € 1.31; H20 = € 1.75; H40 = 2.18; H60 = € 2.61; and BIO = € 2.53. Eurasian perch sale price (SP) was calculated at € 6.50 kg^−1^.

Fish-in fish-out (FIFO) ratio was used as a practical measure of the quantity of live fish from capture fisheries required for each unit of farmed fish produced [31]. This indicator of environmental sustainability of feeds was calculated as follows:

FIFO = (LFM + LFO)/(YFMw + YFOw) × FCR,

where LFM is level of fishmeal in the diet; LFO is level of fish oil in the diet; YFMw is yield of fishmeal from wild fish; YFOw is yield of fish oil from wild fish; FCR is feed conversion ratio.

We estimated the impact of FM substitution with *H. illucens* larva meal rapported to Metric Tons (MT) on freshwater demand (WD, m^3^/MT), land demand (LD, ha/MT), energy use (EU, GJ/MT) and greenhouse gas production (GWP, kg CO_2_^−eq^). Mean WD, LD and EU for FM, wheat, fish oil, starch and mineral and vitamin mixes were obtained from Chatvijitkul et al. [32]. Data of WD, LD, EU and GWP for *H. illucens* larva meal was retrieved from Roffeis et al. [33]. Finally, GWP for FM was sourced from Thevenot et al. [34] and GWP for wheat meal from Heusala et al. [35].

### 2.8. Statistical Analyses

All data were tested for homogeneity of variance using Cochran, Hartley and Bartlett tests. Normality of data was tested by Shapiro-Wilk test. Perivisceral fat index, splenosomatic index, some minor fatty acids, hemoglobin, mean corpuscular hemoglobin, mean corpuscular hemoglobin concentration and economic profit index were analyzed using Kruskal-Wallis non-parametric test as these data does not show normality. All other remaining parameter results were analyzed separately by one-way ANOVA. Differences were considered significant at *p* ≤ 0.05 (post-hoc test: Tukey test). The data were expressed as mean ± SD and statistical analyses were performed using STATISTICA 12.0 (StatSoft CR, Prague, Czech Republic). As BIO was a completely different diet, not comparable with respect to composition, nutrient and energy contents, it was not included in the statistical analyses.

## 3. Results

### 3.1. Diet Composition

Diets were comparable in proximate composition, which reflected the calculated one. The amino acid profile of H. *illucens* larva meal and experimental diets is presented in Table 2. Leucine, tyrosine and valine were the most common EAAs in the H. *illucens* larva meal, with the non-essential AAs glutamic and aspartic acid showing the highest content. *Hermetia illucens* larva meal showed similar values for histidine and lower values for arginine and lysine than observed in FM [36]. With increasing dietary H. *illucens* proportions, all EAAs decreased except valine and tyrosine, which remained constant and increased, respectively.

### 3.2. Growth Trial

Fish survival and growth performance are shown in Table 4. With all diets, fish tripled their initial body weight. Fish readily accepted the feeds and no rejection was recorded. At the end of the 84-day experiment, no significant differences in survival were observed among diets. There were no significant differences among experimental groups in BWi, ICV and FCV. On the other hand, BWf, SGR, PER and FR differed significantly with diet, with the H60 treatment showing lower values compared to other treatments.

### 3.3. Condition Factor, Somatic Indices and Occurrence of Spleen Lipidosis

Fish fed H60 showed lower K and HSI compared to fish fed the CON diet, while no differences among treatments were recorded in any other parameter (Table 5). No splenic lipidosis was recorded in fish fed insect meal, while a high occurrence was recorded in fish fed the BIO diet.

### 3.4. Proximate and Fatty Acid Composition of Whole Fish Homogenate

The proximate composition of the whole fish homogenates showed no significant differences in DM, CP and EE content (Table 6). On the other hand, OM and GE content showed a decreasing trend with increased the *H. illucens* meal in the feed, while the opposite was recorded for ash content.

The fatty acid composition of Eurasian perch was significantly affected by the feed (Table 7). In general, saturated fatty acids (SFA) content tended to increase with increased *H. illucens* larva meal proportions with exception of C15:0 and C20:0. A trend similar to SFA was observed for monounsaturated fatty acids (MUFA) and polyunsaturated fatty acids (PUFA). Significant differences were found both in omega-6 and omega-3 content that decreased among tested *H. illucens* larva meal diets and consequently, the n-3/n-6 ratio decreased with increasing *H. illucens* larva meal inclusion.

### 3.5. Haematological Analyses

Hemoglobin (Hb) concentration, HCT, RBBC, MCV, MCH and MCHC showed no differences among the feeding groups (Table 8).

### 3.6. Economic Analysis and Environmental Sustainability of Feeds

The FIFO index decreased proportionally with increased insect meal proportions, reaching 3.04 (CON), 2.17 (H20), 1.56 (H40) and 1.18 (H60). The H. *illucens* meal diets differed significantly with respect to ECR and EPI (Table 9), with cost increasing concurrent with H. *illucens* meal replacement. The inclusion of insect meal led to an overall increase of environmental sustainability parameters GWP, EU and LD and a reduction in freshwater demand.

## 4. Discussion

Intensive culture of Eurasian perch is still a young industry in Europe with the main producers being Ireland, France, Poland, Belgium and Denmark. Insects have been proposed as an efficient and high-quality alternative protein source for poultry [37,38], swine [11,39] and carnivorous fish [13,15,16,17,20,40] and interest in use of insect meals in perch diets is high. Insects are a viable source of protein and lipids [9,10] and a typical component of Eurasian perch natural diet. Nogales-Mérida [9], confirmed insects as an excellent source of several vitamins and minerals including iron, potassium, calcium and magnesium. Use of H. *illucens* insect meal is consistent with production of perch as an organic product, as insect meal can be produced locally on a variety of substrates [41,42].

The present study represents the first reported use of defatted black soldier fly H. *illucens* larva meal as an alternative feed ingredient for Eurasian perch reared in intensive culture. Bußler et al. [43] demonstrated that H. *illucens* is an appropriate insect species for insect meal production. It has a well-balanced essential amino acid profile, an average protein content of 55% DM and ~35% fat DM, which may be reduced to 5–9% by defatting, making it more digestible. However, complete FM replacement by insect meal has not been shown feasible. Henry et al. [44], reported that the maximum dietary replacement of FM by H. *illucens* meal ranges from 6 to 25%, depending on fish species, with higher inclusion levels reducing growth performance. Sealey et al. [45], reported up to 50% H. *illucens* inclusion without negative effects on growth of rainbow trout. Our study showed that there is no significant effect up to 400 g/kg of H. *illucens* in the perch diet on body weight or specific growth rate. Similar results were demonstrated by Renna et al. [15], where partially defatted H. *illucens* larva meal up to 40% of inclusion level was used in rainbow trout diet without negative effects on survival rate, growth performance, condition factor, somatic indices, physical quality or gut morphology. Magalhaes et al. [17], replaced 45% of the FM in diet of juvenile European seabass with up to 19.5% H. *illucens* meal corresponding to 22.5% protein without adverse effects on growth performance and feed utilization. Kroeckel et al. [20], reported that inclusion higher than 33% of defatted H. *illucens* larvae decreased protein digestibility, feed acceptance and growth performance of juvenile turbot. Lock et al. [12], showed that drying slightly defatted H. *illucens* meal (255 g/kg DM) at low temperature is the most suitable procedure and produced a good alternative feed for Atlantic salmon growth.

Proximate composition of fish is driven by endogenous (size, life cycle stage) as well as exogenous factors (water quality, feed) [46]. To minimize bias, we reared European perch under similar conditions. We found no significant differences in DM, CP and EE in whole-fish homogenate among tested H. *illucens* diets. This is in line with Gasco et al. [47], who found no significant difference in DM and CP content of European sea bass fed mealworm *Tenebrio molitor* at different diet proportions. Contrary results were obtained in rainbow trout fed T. *molitor*, in which increasing the proportion of insect meal triggered significant decreases in DM, CP and EE [48], while increased enriched H. *illucens* prepupae content resulted in decline in DM and EE [45].

Reduction in DM and EE content may result from decreased nutrient availability [15], depending on insect species [23,42] or on its culture substrate [37,39]. Culture substrate also substantially affects insect ash content [49,50]. Although body ash content has been reported similar among fish consuming various insect meal diets [20,51], we found a significant difference among our diet groups, with the highest ash content in H60, while lower ash levels were observed in CON, H20 and H40 groups. This is in contrast to the proximate analysis of tested diets per se, in which the ash content decreased with increasing H. *illucens* inclusion. Kirchgessner and Schwarz [52] and Shearer [46] reported no effect of crude dietary ash on ash content of fish body, provided sufficient levels of essential elements are present. This suggests that the partially defatted H. *illucens* meal used in our study may lack some essential element or elements, although this complex mechanism is largely unexplored and needs further study. The GE content decreased significantly with increased *H. illucens* larva meal inclusion, reflecting the non-significant decrease in both CP and EE with higher *H. illucens* larva content.

We found total n-3 and n-6 fatty acid in Eurasian perch to decrease significantly with higher levels of *H. illucens* larva meal in the diet, reflecting lower fish oil content, with the n-3/n-6 ratio being inversely related to H. *illucens* inclusion. This is in agreement with findings of Borgogno et al. [51] and Renna et al. [15], who reported significant reduction of n-3/n-6 ratio in rainbow trout fed with *H. illucens* larva meal. The opposite effect was observed in Atlantic salmon fed H. *illucens* meal [13]. The differences among studies could be related to diet composition. In the present study, as well as those of Borgogno et al. [53] and Renna et al. [15], fish oil was used as a fat source, while Belghit et al. [54], used large quantities of rapeseed oil, which contain high level of n-6 polyunsaturated fatty acids contributing to maintain constant the n-3/n-6 ratio between insect meal based diets.

These comparisons underscore differences among insect species and culture media. We found increased H. *illucens* proportions to be associated with significantly higher SFA content in fish homogenate, reflecting that partially defatted H. *illucens* meal is rich in SFAs (lauric acid C12:0, myristic acid C14:0 and palmitic acid C16:0), while T. *molitor* larva meal is rich in MUFAs and n-6 PUFAs. A similar trend was observed in studies of Jian carp [55] and rainbow trout [13], fed H. *illucens* larva meal. The positive effect on HSI observed in the present study could be related to reduction of lipid storage in liver, as was demonstrated in Atlantic salmon [54].

Hematological parameters, essential tools in evaluation of fish welfare related to stress and immune status [56,57,58], are highly influenced by feeding regime [59]. Studies of FM substitutes such as cottonseed [60], soybean [61,62], housefly (*Musca domestica)* maggot [63] and cricket (*Gryllus bimaculatus)* [64], showed no significant effect of tested meals on hematological parameters of fish of various species. This reinforces our suggestion that dietary H. *illucens* larva meal does not impact welfare of Eurasian perch but further investigations of diet formulations and feeding strategies are needed to collect additional data for this new area of study and to obtain more comprehensive results on fish growth rate.

The fish-in fish-out ratio is a practical indicator of environmental sustainability [31]. This index uses a global average wet weight (whole fish) to fishmeal yield of 22.5% and wet weight to fish oil yield of 5%. A ratio >1 indicates net removal of fish globally. We found the FIFO ratio to be substantially reduced with increasing proportions of insect meal and that FIFO could be decreased by 49% in perch fed an insect-based diet without affecting growth. This downward trend is in agreement with forecast of Tacon and Marc [65].

Increasing H. *illucens* larva meal proportions in commercial fish feeds could lead to higher energy and land use and increased greenhouse gas production. A lower impact was found for freshwater use. Insect meal inclusion level, which does not affect growth parameters, led to a 144% increase in greenhouse gas production, 123% increase in energy demand and 77% increase in land use. Fresh water use was decreased by 38% compared to control. These findings suggest ongoing monitoring of agricultural resources and related socio-economic and environmental impact during the shift in resource demands from the oceans onto the land.

Future studies should be focused on fine-tuning for optimal insect meal inclusion in the range of 40% to 60%, as well as evaluation of diets with a higher contribution of plant-based protein in combination with insect meal. Long-term studies of rearing fish to a higher market size (>200 g), in combination with sensory and texture analysis of the final product, should be carried out to explore full potential of insect-based diets for perch.

When the inclusion level was >60%, growth was significantly reduced compared with the control group, suggesting that incorporation of up to 40% H. *illucens* larva meal in the feed formulation for perch is feasible and can reduce reliance on marine resources. However, even if presents limitations, such as production cost and increased impact in some environment-related parameters, the partial replacement of fishmeal by insect protein will be more important in the future as getting enough amount of fishmeal will be difficult and culture of insects like a *H*. *illucens* using waste food means to convert non-resources to important protein resources is a promising solution to cope this problem.

## Figures and Tables

**Table 1:** Ingredients and proximate composition of *Hermetia illucens* larva meal and experimental diets.

Ingredients (g/kg)	*H. illucens* Larva Meal	CON	H20	H40	H60
FM (Chile, super prime) ^a^	-	720	570	420	270
*H. illucens* larva meal ^b^		0	200	400	600
Wheat meal	-	120	90	60	30
Fish oil	-	60	40	20	0
Starch, D500	-	80	80	80	80
Mineral mixture ^c^	-	10	10	10	10
Vitamin mixture ^d^	-	10	10	10	10
Proximate composition^e^					
DM (g/100 g)	94.18	88.74	90.76	90.59	90.51
CP (g/100 g DM)	55.34	54.50	54.37	54.10	53.91
EE (g/100 g DM)	17.97	11.92	11.95	11.62	11.64
Ash (g/100 g DM)	7.12	14.77	13.70	12.44	11.41
Chitin (g/100 g DM)	5.00	-	0.98	2.12	3.15
NFE (g/100 g DM) ^f^	14.57	18.81	19.02	19.72	19.89
Gross energy (MJ/kg DM) ^g^		22.90	22.54	23.02	23.26

FM, fishmeal; DM, dry matter; CP, crude protein; EE, ether extract; NFE, nitrogen free extracts, groups CON, H20, H40 and H60 represent 0%, 20%, 40% and 60% inclusion of H. *illucens* meal, respectively. ^a^ Fishmeal was purchased from Corpesca S.A. (Santiago, Chile). Proximate composition (% as-fed basis): 90.4 DM; 66.7 CP; 8.3 EE; 14.9 Ash. ^b^ *Hermetia illucens* larvae meal purchased from Hermetia Deutschland GmbH & Co. KG (Baruth/Mark, Germany). ^c^ Mineral mixture (g or mg/kg diet): bicalcium phosphate 500 g, calcium carbonate 215 g, sodium salt 40 g, potassium chloride 90 g, magnesium chloride 124 g, magnesium carbonate 124 g, iron sulphate 20 g, zinc sulphate 4 g, copper sulphate 3 g, potassium iodide 4 mg, cobalt sulphate 20 mg, manganese sulphate 3 g, sodium fluoride 1 g (Granda Zootecnica, Cuneo, Italy). ^d^ Vitamin mixture (IU or mg/kg diet): DL-tocopherolacetate, 60 IU; sodium menadione bisulphate, 5 mg; retinylacetate, 15,000 IU; DL-cholecalciferol, 3000 IU; thiamine, 15 mg; riboflavin, 30 mg; pyridoxine, 15 mg; vitamin B12, 0.05 mg; nicotinic acid, 175 mg; folic acid, 500 mg; inositol, 1000 mg; biotin, 2.5 mg; calcium panthotenate, 50 mg; choline chloride, 2000 mg (Granda Zootecnica, Cuneo, Italy). ^e^ Values are reported as mean of triplicate analyses. ^f^ Calculated as 100 − (CP + EE + Ash + Chitin). ^g^ Determined by bomb calorimetry.

**Table 2:** Amino acid (AA) profile (% of protein) of *Hermetia illucens* larva meal and experimental diets.

	*H. illucens*	CON	H20	H40	H60
**Essential AA**					
Arginine	3.9	6.2	5.7	5.2	4.7
Histidine	2.2	2.4	2.4	2.3	2.3
Isoleucine	3.3	4.2	4.0	3.8	3.6
Leucine	5.2	7.3	6.8	6.4	5.9
Lysine	3.8	7.4	6.7	5.9	5.1
Methionine	2.1	2.7	2.5	2.2	2.0
Cysteine	0.1	0.9	0.7	0.5	0.4
Phenylalanine	3.0	4.0	3.7	3.5	3.3
Tyrosine	4.8	3.1	3.4	3.8	4.1
Threonine	3.1	4.1	3.9	3.7	3.5
Valine	4.9	4.9	4.9	4.9	4.9
**Non-essential AA**					
Alanine	6.2	6.1	6.1	6.1	4.9
Aspartic acid	6.7	8.8	8.4	7.9	7.5
Glycine	4.2	0.9	1.6	2.2	2.9
Glutamic acid	8.8	7.0	7.3	7.6	7.9
Proline	5.5	12.3	10.9	9.5	8.0
Serine	3.7	4.1	4.0	3.9	3.8

CON, H20, H40 and H60 represent 0%, 20%, 40% and 60% inclusion of H. *illucens* larva meal, respectively.

**Table 3:** Fatty acid profile of experimental diets for Eurasian perch. Data are expressed as percent of total FAs (mean ± SD, n = 3).

FA	CON	H2O	H4O	H6O
C12:0	2.54 ± 0.27	11.89 ± 0.17	24.47 ± 1.81	34.37 ± 1.23
C14:0	4.76 ± 0.04	6.54 ± 0.06	8.77 ± 0.11	10.74 ± 0.38
C14:1	0.05 ± 0.01	0.13 ± 0.01	0.24 ± 0.01	0.33 ± 0.01
C15:0	0.40 ± 0.01	0.35 ± 0.01	0.28 ± 0.01	0.23 ± 0.01
C16:0	15.38 ± 0.04	16.01 ± 0.05	16.58 ± 0.32	17.21 ± 0.69
C16:1	4.21 ± 0.01	4.49 ± 0.01	4.79 ± 0.10	5.08 ± 0.11
C18:0	3.77 ± 0.03	3.63 ± 0.04	3.22 ± 0.07	3.02 ± 0.13
C18:1n9trans	0.08 ± 0.01	0.07 ± 0.01	0.07 ± 0.01	0.06 ± 0.01
C18:1n9	26.60 ± 0.20	22.08 ± 0.02	16.20 ± 0.36	11.29 ± 0.27
C18:1n7	3.23 ± 0.02	2.57 ± 0.01	1.68 ± 0.04	0.94 ± 0.04
C18:2n6	9.18 ± 0.03	8.61 ± 0.01	7.79 ± 0.18	7.20 ± 0.14
C18:3n6	0.20 ± 0.01	0.16 ± 0.01	0.11 ± 0.01	0.07 ± 0.01
C18:3n3	3.15 ± 0.01	2.56 ± 0.01	1.80 ± 0.05	1.14 ± 0.03
C20:0	0.32 ± 0.01	0.27 ± 0.01	0.21 ± 0.01	0.15 ± 0.01
C20:1n9	2.55 ± 0.02	1.91 ± 0.01	1.05 ± 0.03	0.32 ± 0.03
C20:3n6	0.76 ± 0.01	0.56 ± 0.01	0.31 ± 0.01	0.09 ± 0.01
C20:3n3	0.71 ± 0.01	0.56 ± 0.01	0.38 ± 0.01	0.23 ± 0.01
C20:4n6	0.32 ± 0.01	0.24 ± 0.01	0.13 ± 0.01	0.03 ± 0.01
C22:0	0.16 ± 0.01	0.13 ± 0.01	0.08 ± 0.01	0.05 ± 0.01
C22:1n9	0.37 ± 0.01	0.27 ± 0.01	0.15 ± 0.01	0.04 ± 0.01
C20:5n3	6.80 ± 0.03	5.44 ± 0.01	3.72 ± 0.10	2.21 ± 0.01
C22:2	0.07 ± 0.01	0.05 ± 0.01	0.32 ± 0.50	0.65 ± 0.56
C24:0	0.17 ± 0.01	0.13 ± 0.01	0.10 ± 0.01	0.05 ± 0.03
C24:1n9	0.55 ± 0.01	0.44 ± 0.01	0.29 ± 0.01	0.16 ± 0.01
C22:5n3	1.44 ± 0.01	1.06 ± 0.02	0.69 ± 0.02	0.31 ± 0.02
C22:6n3	12.23 ± 0.18	9.85 ± 0.11	6.61 ± 0.15	4.03 ± 0.14
SFA	27.58 ± 0.34	39.00 ± 0.12	54.02 ± 1.06	66.47 ± 0.77
MUFA	37.63 ± 0.23	31.96 ± 0.03	24.45 ± 0.54	18.23 ± 0.40
PUFA	34.79 ± 0.21	29.04 ± 0.11	21.53 ± 0.51	15.30 ± 0.38
n-3	24.33 ± 0.22	19.47 ± 0.12	13.20 ± 0.32	7.90 ± 0.28
n-6	10.46 ± 0.03	9.57 ± 0.01	8.33 ± 0.19	7.39 ± 0.15
n-3/n-6	2.33 ± 0.02	2.03 ± 0.01	1.58 ± 0.01	1.07 ± 0.03

CON, H20, H40 and H60 represent 0%, 20%, 40% and 60% inclusion of *H. illucens* larva meal, respectively; SD, standard deviation, FA fatty acid, SFA saturated fatty acids, MUFA, monounsaturated fatty acids, PUFA, polyunsaturated fatty acids.

**Table 4:** Survival and growth performance of Eurasian perch fed experimental diets and the commercial control diet (mean ± SD; n = 3).

Items	CON	H20	H40	H60	SEM	*p*-Value	BIO *
Survival, %	98.7 ± 2.3	98.7 ± 2.3	98.0 ± 1.2	99.3 ± 1.2	0.512	0.878	96.0 ± 4.0
BW_i_, g	21.9 ± 0.1	22.0 ± 0.1	22.1 ± 0.1	22.0 ± 0.1	0.023	0.195	22.0 ± 0.1
BW_f_, g	63.8 ± 1.2 ^a^	67.1 ± 2.0 ^a^	68.1 ± 1.8 ^a^	58.0 ± 3.2 ^b^	1.305	0.002	74.1 ± 6.0
WG, g	41.8 ± 1.0 ^a^	45.1 ± 2.0 ^a^	46.0 ± 1.7 ^a^	36.0 ± 3.2 ^b^	1.296	0.002	52.1 ± 5.9
ICV, %	19.4 ± 0.6	19.6 ± 0.8	19.5 ± 0.9	19.3 ± 0.9	0.205	0.981	19.0 ± 0.9
FCV, %	37.9 ± 1.6	32.9 ± 7.0	34.3 ± 7.1	38.3 ± 1.8	1.439	0.525	42.4 ± 9.1
SGR, %/d	1.25 ± 0.06 ^a,b^	1.30 ± 0.03 ^a^	1.30 ± 0.04 ^a^	1.14 ± 0.03 ^b^	1.331	0.000	1.39 ± 0.11
FCR	1.00 ± 0.07 ^a,b^	0.91 ± 0.05 ^b^	0.91 ± 0.04 ^b^	1.12 ± 0.06 ^a^	0.029	0.006	0.96 ± 0.13
PER	1.72 ± 0.12 ^a,b^	1.91 ± 0.11 ^a^	1.90 ± 0.08 ^a^	1.55 ± 0.08 ^b^	0.050	0.000	1.88 ± 0.23
FR, %/d	1.36 ± 0.03 ^a,b^	1.30 ± 0.04 ^a^	1.30 ± 0.01^a^	1.39 ± 0.04 ^b^	0.014	0.023	1.47 ± 0.06

CON, H20, H40 and H60 represent 0%, 20%, 40% and 60% inclusion of *H. illucens* larva meal respectively; BIO is a commercial diet (BioMar Inicio, Brande, Denmark). SD, standard deviation, SEM, standard error of the mean; BW_i_, initial body weight; BW_f_, final body weight; WG, weight gain; ICV, initial coefficient of variation of weight; FCV, final coefficient of variation of weight; SGR, specific growth rate; FCR, feed conversion ratio; PER, protein efficiency ratio; FR, feeding rate. Different letters within a row indicate significant differences (*p* ≤ 0.05). * Statistical analysis did not include BIO.

**Table 5:** Condition factor (n = 45), somatic indices and occurrence of splenic lipidosis (n = 90) of Eurasian perch juveniles fed experimental diets and the commercial control diet (mean ± SD).

Items	CON	H20	H40	H60	SEM	*p*-Value	BIO *
K	1.20 ± 0.02 ^a,b^	1.22 ± 0.02 ^a^	1.19 ± 0.01 ^a,b^	1.15 ± 0.01 ^b^	0.008	0.020	1.28 ± 0.03
HSI	1.76 ± 0.20 ^a^	1.41 ± 0.12 ^a,b^	1.48 ± 0.10 ^a,b^	1.21 ± 0.07 ^b^	0.067	0.006	1.37 ± 0.04
SSI	0.12 ± 0.04	0.11 ± 0.04	0.10 ± 0.04	0.11 ± 0.05	0.010	0.964	0.13 ± 0.02
VSI	2.91 ± 0.38	2.79 ± 0.17	2.94 ± 0.19	3.06 ± 0.05	0.063	0.608	2.90 ± 0.19
PFI	6.19 ± 0.68	5.63 ± 0.19	6.06 ± 0.78	5.53 ± 0.25	0.157	0.826	9.43 ± 0.97
SL	3.9 ± 6.71	NF	NF	NF		-	19.5 ± 13.6

CON, H20, H40 and H60 represent 0%, 20%, 40% and 60% inclusion of *H. illucens* larva meal, respectively, BIO is a commercial diet (BioMar Inicio, Brande, Denmark). SD, standard deviation, SEM, standard error of the mean; K, condition factor; HSI, hepatosomatic index; SSI, splenosomatic index; VSI, visceromatic index; PFI, perivisceral fat index; SL, splenic lipidosis; NF, not found. Different letters within a row indicate significant difference (*p* ≤ 0.05). * Statistical analysis did not include BIO. No statistical analysis was performed for SL, as some diet groups did not show lipidosis.

**Table 6:** Proximate composition of whole-body homogenate of Eurasian perch fed experimental diets and the commercial control diet (mean ± SD, n = 6).

Items	CON	H20	H40	H60	SEM	*p*-Value	BIO *
DM (g/100 g)	33.3 ± 1.0	32.9 ± 0.6	32.5 ± 0.6	32.1 ± 0.5	0.186	0.142	36.6 ± 1.1
CP (g/100 g DM)	24.1 ± 3.1	21.8 ± 0.9	21.6 ± 0.6	20.7 ± 0.3	0.466	0.065	22.4 ± 0.5
EE (g/100 g DM)	10.1 ± 1.3	9.5 ± 0.2	8.7 ± 0.5	8.5 ± 0.8	0.232	0.052	13.5 ± 1.0
OM (g/100 g DM)	28.6 ± 1.0 ^a^	27.9 ± 0.6 ^a^	27.2 ± 0.6 ^a^	26.4 ± 0.8 ^b^	0.254	0.001	32.1 ± 0.9
Ash (g/100 g DM)	4.7 ± 0.3 ^b^	5.0 ± 0.2 ^b^	5.3 ± 0.2 ^b^	5.6 ± 0.3 ^a^	0.098	0.003	4.5 ± 0.4
GE (MJ/kg DM)	0.81 ± 0.04 ^a^	0.78 ± 0.01 ^a^	0.75 ± 0.02 ^a^	0.74 ± 0.03 ^b^	0.009	0.014	0.95 ± 0.04

CON, H20, H40 and H60 represent 0%, 20%, 40% and 60% inclusion of *H. illucens* larva meal, respectively; BIO is a commercial diet (BioMar Inicio, Brande, Denmark). SD, standard deviation, SEM, standard error of the mean; DM, dry matter; CP, crude protein; EE, ether extract; OM, organic matter; GE, gross energy. Different letters within a row indicate significant difference (*p* ≤ 0.05). * Statistical analysis did not include BIO.

**Table 7:** Fatty acid (FA) profile of whole-body homogenate Eurasian perch fed experimental and commercial control diet. Data are expressed as percent of total FAs (mean ± SD, n = 9).

FA	CON	H20	H40	H60	SEM	*p*-Value	BIO *
C12:0	1.00 ± 1.56 ^b^	4.76 ± 1.81 ^b^	8.83 ± 1.24 ^a,b^	12.03 ± 1.49 ^a^	0.79	<0.001	0.20 ± 0.20
C14:0	4.67 ± 0.61 ^d^	5.96 ± 0.64 ^c^	7.41 ± 0.35 ^b^	9.14 ± 0.72 ^a^	0.315	<0.001	5.52 ± 0.18
C14:1	0.54 ± 0.07 ^d^	0.63 ± 0.05 ^c^	0.79 ± 0.03 ^b^	0.96 ± 0.06 ^a^	0.03	<0.001	0.66 ± 0.03
C15:0	0.42 ± 0.02 ^a^	0.36 ± 0.03 ^a,b^	0.34 ± 0.02 ^b^	0.34 ± 0.05 ^b^	0.008	<0.002	0.49 ± 0.02
C16:0	17.49 ± 1.04	18.45 ± 0.79	17.81 ± 0.67	18.3 ± 1.79	0.206	0.335	18.98 ± 1.03
C16:1	9.46 ± 0.57 ^b^	9.9 ± 0.46 ^a,b^	10.35 ± 0.42 ^a^	10.44 ± 0.51 ^a^	0.109	0.001	10.81 ± 0.41
C18:0	1.6 ± 0.07	1.20 ± 0.74	0.96 ± 0.80	1.70 ± 0.40	0.112	0.262	1.23 ± 0.08
C18:1n9trans	1.06 ± 0.10 ^a^	0.84 ± 0.16 ^b^	0.84 ± 0.12 ^b^	0.74 ± 0.13 ^b^	0.03	<0.001	2.57 ± 0.13
C18:1n9	27.34 ± 0.96 ^a^	26.34 ± 0.79 ^a^	23.99 ± 1.04 ^b^	21.42 ± 0.68 ^c^	0.436	<0.001	21.16 ± 0.36
C18:1n7	3.07 ± 0.20 ^a^	2.00 ± 1.24 ^a,b^	1.70 ± 1.05 ^b^	2.07 ± 0.17 ^a^	0.167	<0.001	2.76 ± 0.08
C18:2n6	8.21 ± 0.40 ^a^	7.74 ± 0.28 ^a,b^	7.79 ± 0.40 ^a^	7.28 ± 0.40 ^b^	0.087	<0.001	7.15 ± 0.24
C18:3n6	0.08 ± 0.09 ^a,b^	0.06 ± 0.08 ^b^	0.04 ± 0.07 ^b^	0.17 ± 0.01 ^a^	0.015	0.005	0.15 ± 0.01
C18:3n3	2.23 ± 0.16 ^a^	1.99 ± 0.09 ^b^	1.65 ± 0.08 ^c^	1.30 ± 0.07 ^d^	0.065	<0.001	1.77 ± 0.05
C20:0	1.02 ± 0.38 ^a^	1.07 ± 0.04 ^a^	0.97 ± 0.09 ^a^	0.42 ± 0.37 ^b^	0.065	0.001	0.98 ± 0.95
C20:1n9	2.21 ± 0.23 ^a^	1.73 ± 0.23 ^a,b^	1.58 ± 0.22 ^b^	1.64 ± 0.36 ^b^	0.063	0.003	4.25 ± 0.16
C20:3n6	0.11 ± 0.05 ^a^	0.07 ± 0.06 ^a,b^	0.06 ± 0.04 ^b^	0.09 ± 0.02 ^a,b^	0.008	0.035	0.06 ± 0.03
C20:3n3	0.38 ± 0.25 ^a^	0.32 ± 0.20 ^a^	0.18 ± 0.19 ^b^	0.30 ± 0.04 ^a^	0.034	0.050	0.41 ± 0.04
C20:4n6	0.19 ± 0.08 ^a^	0.09 ± 0.10 ^a,b^	0.05 ± 0.07 ^b^	0.11 ± 0.03 ^a,b^	0.015	0.009	0.14 ± 0.02
C22:0	nd	nd	nd	nd			nd
C22:1n9	nd	nd	nd	nd			nd
C20:5n3	0.55 ± 0.47	0.58 ± 0.26	0.74 ± 0.14	0.56 ± 0.36	0.057	0.629	1.06 ± 1.47
C22:2	4.23 ± 0.39 ^a^	3.58 ± 0.33 ^b^	3.14 ± 0.38 ^b^	2.62 ± 0.34 ^c^	0.122	<0.001	5.99 ± 0.18
C24:0	nd	nd	nd	nd			nd
C24:1n9	nd	nd	nd	nd			nd
C22:5n3	1.16 ± 0.15 ^a^	0.98 ± 0.12 ^b^	0.75 ± 0.07 ^c^	0.58 ± 0.06 ^d^	0.043	<0.001	1.09 ± 0.09
C22:6n3	12.91 ± 1.2 ^a^	11.35 ± 1.12 ^b^	10.04 ± 0.60 ^b^	7.66 ± 0.98 ^c^	0.385	<0.001	12.46 ± 0.59
SFA	30.43 ± 2.54 ^d^	35.37 ± 2.38 ^c^	39.47 ± 1.38 ^b^	44.62 ± 2.55 ^a^	1.012	<0.001	33.39 ± 1.39
MUFA	43.74 ± 0.98 ^a^	41.44 ± 0.89 ^b^	39.25 ± 0.61 ^c^	37.34 ± 1.01 ^d^	0.455	<0.001	42.31 ± 0.90
PUFA	25.82 ± 1.94 ^a^	23.18 ± 1.77 ^b^	21.28 ± 1.00 ^b^	18.04 ± 1.70 ^c^	0.579	<0.001	24.30 ± 1.67
n-3	17.23 ± 1.58 ^a^	15.22 ± 1.46 ^b^	13.34 ± 0.78 ^c^	10.40 ± 1.35 ^d^	0.504	<0.001	16.80 ± 1.57
n-6	8.59 ± 0.40 ^a^	7.97 ± 0.45 ^b^	7.94 ± 0.33 ^b^	7.64 ± 0.37 ^b^	0.090	<0.001	7.50 ± 0.26
n-3/n-6	2.00 ± 0.12 ^a^	1.91 ± 0.14 ^a^	1.68 ± 0.09 ^b^	1.36 ± 0.12 ^c^	0.049	<0.001	2.24 ± 0.20

CON, H20, H40 and H60 represent 0%, 20%, 40% and 60% inclusion of *H. illucens* larva meal, respectively; BIO is a commercial diet (BioMar Inicio, Brande, Denmark); SD, standard deviation, SEM, standard error of the mean; SFA saturated fatty acids, MUFA, monounsaturated fatty acids, PUFA, polyunsaturated fatty acids. Different letters within a row indicate significant difference (*p* ≤ 0.05). * Statistical analysis did not include BIO; nd = not detected.

**Table 8:** Hematological parameters of Eurasian perch fed experimental and commercial control diet (mean ± SD, n = 9).

Items	Unit	CON	H20	H40	H60	SEM	*p*-Value	BIO *
Hb	(g/L)	51.5 ± 2.9	51.4 ± 2.6	50.5 ± 3.6	51.0 ± 2.8	0.426	0.987	51.8 ± 2.8
HCT	(L/L)	32.4 ± 6.9	31.9 ± 10.2	30.4 ± 6.1	28.8 ± 9.3	1.180	0.829	32.7 ± 7.5
RBBC	(T/L)	1.90 ± 0.3	1.75 ± 0.4	1.58 ± 0.2	1.81 ± 0.6	0.060	0.466	1.89 ± 0.4
MCV	(fl)	173.1 ± 43.4	185.5 ± 46.7	191.5 ± 25.8	165.9 ± 41.5	5.534	0.616	174.1 ± 25.4
MCH	(pg)	27.6 ± 4.5	32.5 ± 4.5	32.4 ± 5.0	33.8 ± 4.9	1.767	0.296	28.7 ± 6.8
MCHC	(g/L)	0.17 ± 0.04	0.18 ± 0.07	0.17 ± 0.04	0.20 ± 0.08	0.009	0.931	0.17 ± 0.05

Groups CON, H20, H40 and H60 represent 0%, 20%, 40% and 60% inclusion, of H. *illucens* meal, respectively; BIO is a commercial diet (BioMar Inicio, Brande, Denmark). SD, standard deviation, Hb, hemoglobin concentration; HCT, hematocrit; RBCC, red blood cell count; MCV, mean corpuscular volume; MCH, mean corpuscular hemoglobin; MCHC, mean corpuscular hemoglobin concentration; SEM, standard error mean; * Statistical analysis did not include BIO.

**Table 9:** Economic and environmental sustainability parameters of European perch production using feeds differing in insect meal inclusion level (mean ± SD, n = 3).

Items	CON	H20	H40	H60	SEM	*p*-Value	BIO *
FIFO	3.04 ± 0.21 ^a^	2.17 ± 0.12 ^b^	1.56 ± 0.07 ^c^	1.18 ± 0.06 ^d^	0.214	< 0.01	-
GWP (kg CO_2_-eq)	1.81	2.64	3.48	4.32	-	-	-
EU (GJ/MT)	15.35	24.80	34.26	43.71	-	-	-
LD (ha/MT)	0.06	0.08	0.11	0.13	-	-	-
WD (m^3^/MT)	376	304	232	161	-	-	-
ECR	1.4 ± 0.10 ^c^	1.71 ± 0.10 ^c^	2.13 ± 0.10 ^b^	3.13 ± 0.17 ^a^	0.198	<0.01	2.62 ± 0.34
EPI	0.36 ± 0.01 ^a^	0.36 ± 0.01 ^a^	0.34 ± 0.01 ^a^	0.26 ± 0.02 ^b^	0.012	<0.04	0.35 ± 0.04

CON, H20, H40 and H60 represent 0%, 20%, 40% and 60% inclusion of H. *illucens* meal, respectively; BIO is a commercial diet (BioMar Inicio, Brande, Denmark). SD, standard deviation, FIFO, fish-in fish-out ratio; ECR, economic conversion ratio; EPI, economic profit index, GWP, global warming potential; EU, energy use; LD, land demand; WD, water demand; Different letters within a row indicate significant difference (*p* ≤ 0.05). * The statistical analysis did not include BIO.
